# Exploring the degrees of distortion in simulated human bite marks

**DOI:** 10.1007/s00414-019-02163-5

**Published:** 2019-11-05

**Authors:** Neha Dama, Andrew Forgie, Scheila Mânica, Gavin Revie

**Affiliations:** 1 Centre for Forensic and Legal Medicine and Dentistry, University of Dundee, 2 Park Place, Dundee, Scotland DD1 4HR UK; 2 Glasgow Dental Hospital and School, University of Glasgow, Scotland, UK

**Keywords:** Bite mark, Touch mark, Distortion, Skin, Posture

## Abstract

The properties of the skin and the posture of the body during photographic recording are factors that cause distortion in the bite mark injury. This study aimed to explore the degree of distortion between a ‘touch mark’ (method 1) and a ‘bite mark’ (method 2) on the left upper arm at three different positions (arm relaxed; arm flexed in two different positions). A pair of dental casts with biting edges coated in ink was used to create a mark in 30 subjects (6 ♂, 24 ♀) aged 20–50 years old. Photographs were taken using a Nikon DX digital camera (D5000). The mesiodistal widths and angle of rotations of both upper right central incisor and lower right central incisor and the inter-canine distances were analysed and compared with the true measurements using Adobe Photoshop CC 2017. Statistical analysis was carried out using SPSS Statistics 22 applying a 2 (mark type) × 3 (position) repeated measures ANOVA. For all measures studied, there was a statistically significant difference between mark types and positions. In the case of bite marks, a great degree of distortion was detected, and this increased further when changing the position of the arm. The findings demonstrated that skin properties and posture influence distortion. This could lead to inaccurate measurements and misleading pattern interpretation of bite mark injuries.

## Introduction

Bite mark analysis is the process where the patterned injury and the circumstances surrounding it are taken into consideration. Comparing the injury to a suspect or from a limited population group is called bite mark comparison [1].

Even though bite mark evidence is accepted in courts, its fundamental validity and scientific basis are frequently challenged. This has resulted in increased scrutiny in courts to ensure its reliability [2]. Moreover, the increase in the number of wrongful convictions based on bite mark evidence has attracted attention from the legal system and advisory bodies who deal with matters of wrongful conviction [3].

In the 1970s, research about human tooth shape variability has occurred in superficial attempts and ‘uniqueness’ became confirmed in court room statements by dentists [4]. Another reasons for uncertainty about ‘the value and scientific validity of comparing and identifying bite marks’ are the unsatisfactory nature of skin as a substrate for registration of tooth impressions (primary distortion) and also the posture of the body (secondary distortion) [5–7]. Several biomechanical properties of the skin contribute to the distortion including non-linearity and viscoelasticity which vary according to the underlying tissue, adherence to musculature, and anatomic location [8]. Postural distortion occurs when the bite mark is photographed in a position different to the position the bite was initially created in [6].

Some reports from the National Academy of Science (NAS) and the President’s Council of Advisors on Science and Technology (PCAST) have highlighted that some forensic methods have never been validated or are clearly invalid. For instance, bite mark identification has been discredited by both scientific studies and false convictions based on the method [9]. The NAS study resulted in the 2009 publication of *Strengthening Forensic Science in the United States: A Path Forward*, which concluded that many forensic tests, including bite marks, have never been exposed to stringent scientific scrutiny [10]. In 2016, a PCAST report concluded that “bitemark analysis does not meet the scientific standards for foundational validity, and is far from meeting such standards. To the contrary, available scientific evidence strongly suggests that examiners cannot consistently agree on whether an injury is a human bitemark and cannot identify the source of bitemark with reasonable accuracy” [11]. In the same year, the Texas Forensic Science Commission recommended bite mark comparison evidence not be admitted in criminal cases in Texas unless some criteria would be established as follows: (a) *criteria for identifying when a patterned injury constitutes a human bite mark*; (b) *criteria for identifying when a human bite mark was made by an adult versus a child*; and (c) *rigorous and appropriately validated proficiency testing* [12]. This study aimed to explore the degree of skin distortion between a ‘touch mark’ and a simulated ‘bite mark’ on the middle third area of the left upper arm at three different positions.

## Materials and methods

This study was ethically approved by the Nursing & Health Sciences and Dentistry Research Ethics Committee (SREC), University of Dundee, UK (Application Number: 2016012_Dama). A number of 30 anonymised students (6 ♂, 24 ♀) of the University of Dundee, Scotland (UK), aged from 20 to 50 years participated in this study. Excluding criteria included skin disorders or current injuries on the arm, bleeding disorders, blood-borne virus infection, and antibiotic use in the previous 2 weeks.

Upper and lower impressions of one of the authors were obtained using polyvinylsiloxane impression material (PVS putty, Zhengzhou, China) and scanned to obtain a digital design file used by the *EnvisionTEC Perfactory DLP printer* to produce the 3D models from *NextDent 3D model liquid* by digital light processing. These models were mounted on an articulator (Denar^®^ MkII, Prestige Dental, Bradford, England) using dental die stone and a vise grip sheet metal plier. Prior to making any marks on skin, the incisal edges of the anterior teeth and 1st and 2nd premolar of dental models were coated with ink by pressing these models on an ink pad (Max Stamp).

In order to locate a standard site to register the mark, the subject was asked to stand upright on both feet with the left arm bent 90° at the elbow joint with the left palm facing in the upward direction. Then, end of the spine of the scapula was located, and using a marker, a horizontal line was drawn on the uppermost edge on the posterior border of the spine extending from the acromion process. A measuring tape was held from the horizontal line drawn at the acromion process and was extended down following the centre of the posterior surface of the arm up to the olecranon process (bony prominence of the mid-elbow), and at this point, another horizontal line was drawn. The distance between these two horizontal lines was measured, and the upper arm was divided into three parts and the teeth marks were created in the middle third of the left upper arm.

**Fig. 1:**
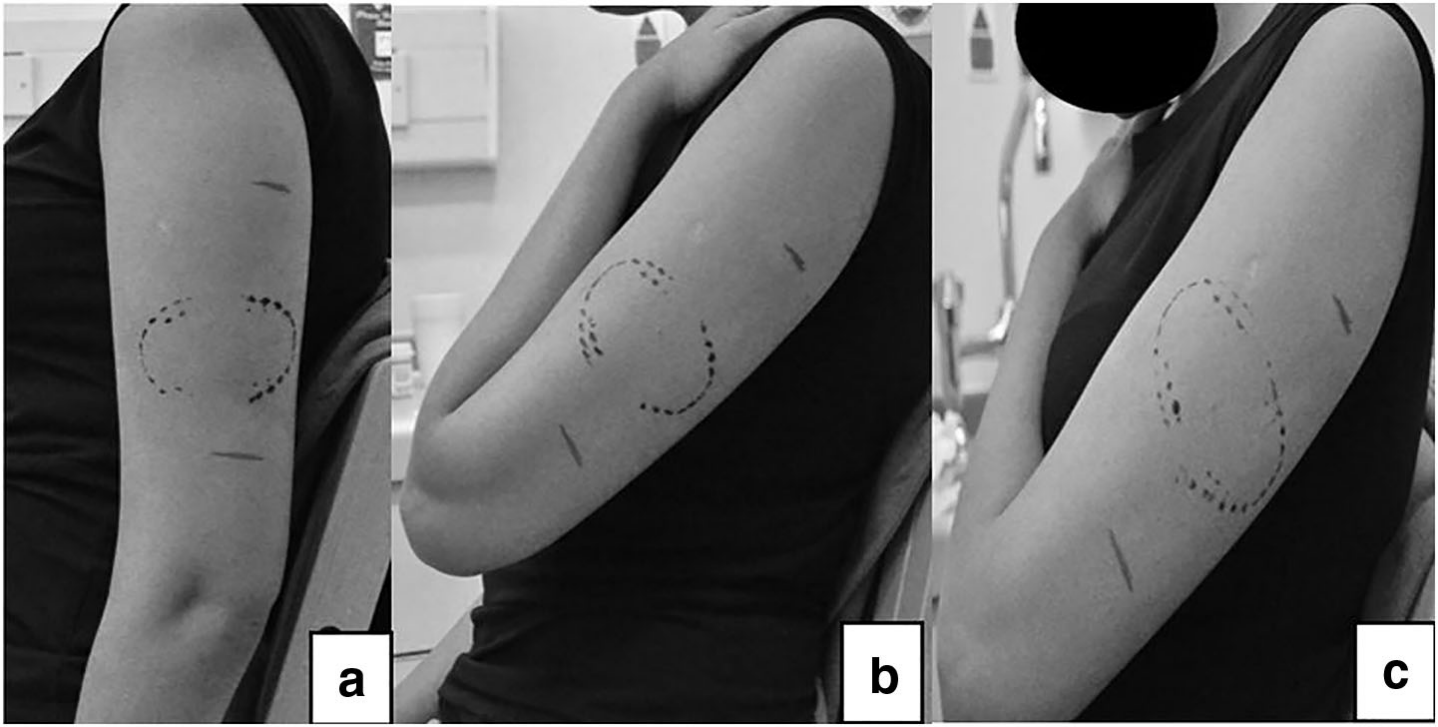
Left arm in three different positions: **a** position 1, **b** position 2, and **c** position 3

Following, the subject was asked to be seated on a chair and the ‘touch mark’ or simulated ‘bite mark’ made. At position 1, the participant was asked to keep the left arm extended position beside the body as shown in Fig. 1 a. A ‘touch mark’ was created on the middle third of the left upper arm with minimal pressure, mimicking the relationship of the upper and lower jaws in human beings (method 1). Photographs were taken using a Nikon DX digital camera (D5000) perpendicular to the mark to reduce the degree of photographic distortion and a ForensiGraph 90° Right Angle scale (ForensiGraph, Essex, UK). To allow more accurate analysis, images were saved (.NEF). The same procedure was repeated for position 2 where the participant flexes his/her left arm and the palm of the left arm rests on the shoulder of the same arm as shown in Fig. 1 b, and to position 3 where the left arm of the participant was held across his/her chest with the palm of his/her left arm rests on his/her opposite shoulder as seen in Fig. 1 c. After at least 1 h, the procedure was repeated on each subject but using the maximum force that the subject could bear in order to create a simulated ‘bite mark’ (method 2). The ‘touch mark’ was used as gold standard dataset.

## Measurements and statistical analysis

**Fig. 2:**
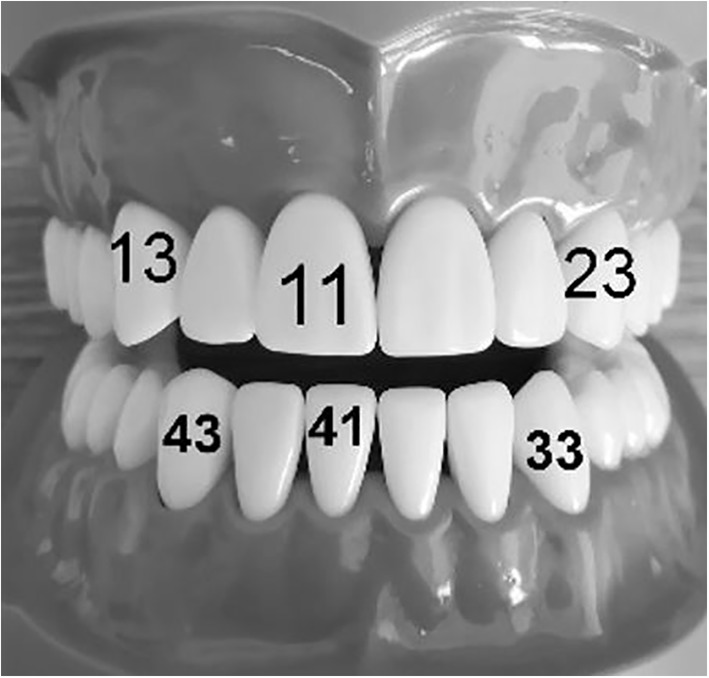
Didactic dental models showing teeth used for metric measurements

The photographs were analysed using Adobe Photoshop CC (2017) to obtain the required metric measurements: the mesiodistal (MD) widths and angle of rotations of both the upper right central incisor (tooth #11) and the lower right central incisor (tooth #41) and the inter-canine distance of upper (distance between the upper right canine [tooth #13] and the upper left canine [tooth #23]) and lower (distance between the lower left canine [tooth #33] and the lower right canine [tooth #43]) arches as seen in Fig. 2. The 3D dental models were scanned, and the real image was stored for the records. A digital copy was obtained to record the true values of those metric measurements.

**Fig. 3:**
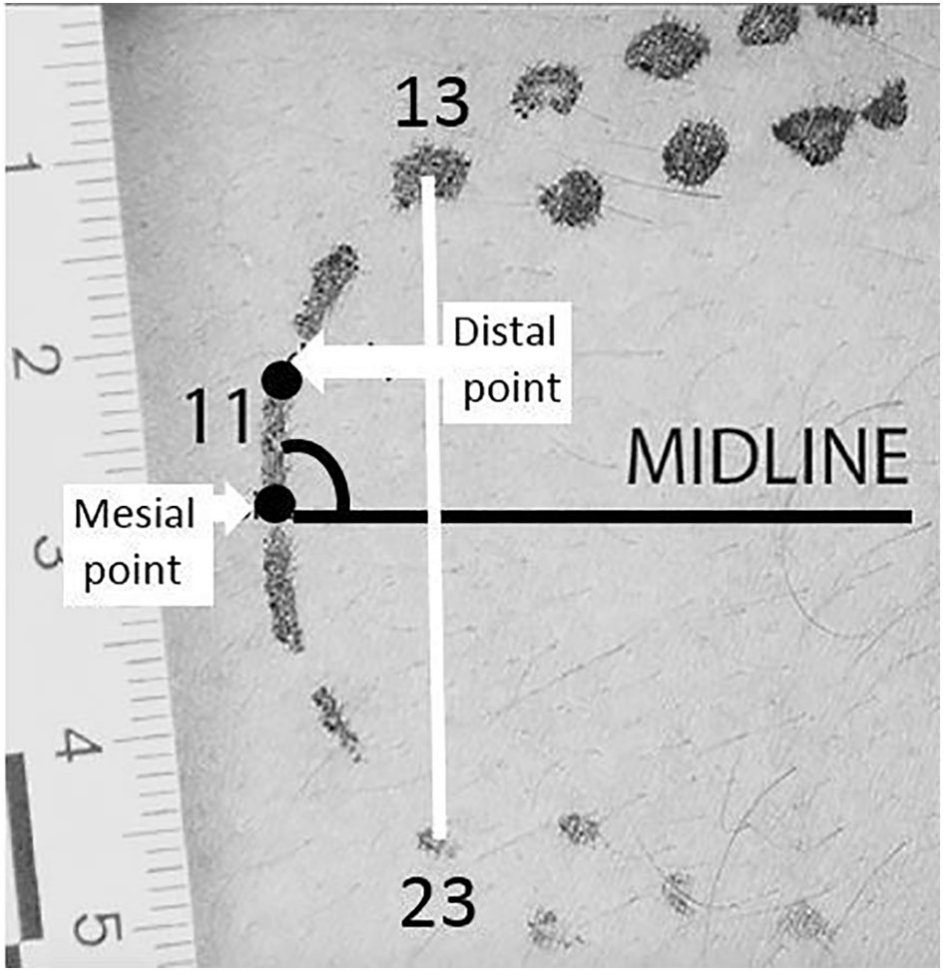
Measurements for upper arch

Mesiodistal width is measured from the mesial point (the point near the midline of the arch) to the most distal point (it is the point away from the midline of the arch) of the incisal edge of a tooth. The inter-canine distance is obtained by first measuring the MD widths of the two canines in the arch; then, the arbitrary midpoints of both the canines are marked, and a line is drawn across the arch joining these two arbitrary midpoints. This distance between the two arbitrary midpoints of the canines is the measured inter-canine. Angle of rotation is measured by first drawing a line from the mesial point to the distal point of a tooth which gives the measure of the MD width. The angle between the midline and line drawn for the MD width of a tooth is the measured angle of rotation. All measurements are shown in Fig. 3.

The data was statistically analysed with a 2 (mark type) by 3 (position) repeated measures ANOVA in SPSS Statistics 22. Intra-class correlation (ICC class 2, single measures, consistency) tests were performed for the intra-rater reliability. The difference in the MD widths (mm) and angle of rotations of #11 and #41 (°) and the inter-canine distance of both arches (mm) at three different positions for both methods were compared with the true measurements. There were two independent variables in this analysis: mark type (touch/bite mark) and position (1, 2, and 3). The dependent variables in this study are the six metric measurements which are each analysed with their own separate ANOVA which will have its results Bonferroni adjusted for having to use 6 ANOVAs*.*

## Analysis of results

**Fig. 4:**
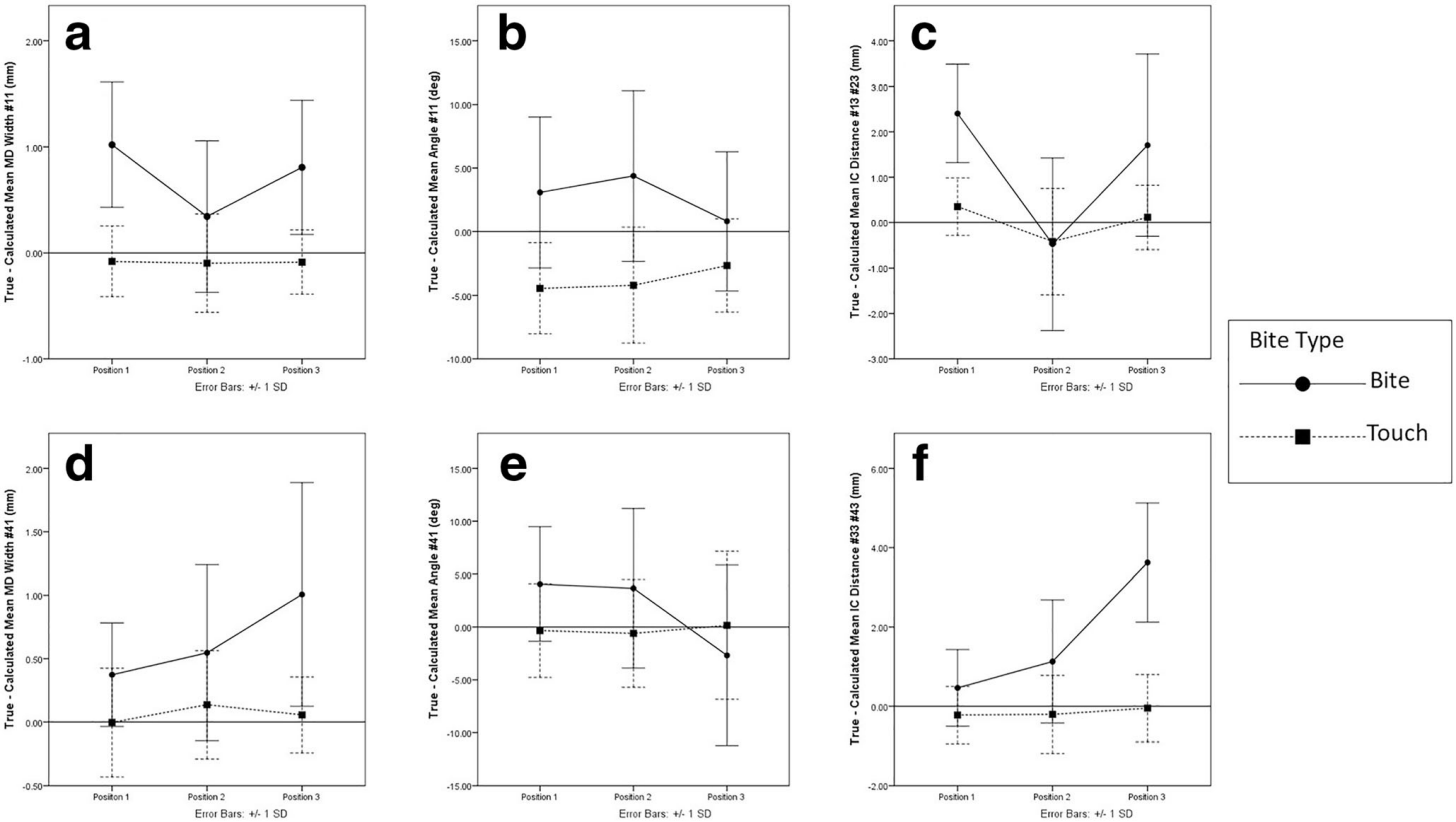
Plot graphs for the six measurements for method 1 (touch mark represented by traced line, square extremes) and method 2 (bite mark represented by solid line, round extremes) and respective standard deviations at three different positions. **a** MD width of tooth #11, **b** angle of rotation of tooth #11, **c** inter-canine distance of the upper arch, **d** MD width of tooth #41, **e** angle of rotation of tooth #41, and **f** inter-canine distance of the lower arch

ICC results for the intra-rater reliability results for MD width of tooth #11 (0.97), MD width of tooth #41 (0.64), angle of rotation of tooth #11 (0.84), angle of rotation of tooth #11 (0.71), and inter-canine distances (0.67 upper and 0.71 lower) were good. The result of the analyses is reported in Table 1. The data for each ANOVA was inspected for outliers and overly influential cases using studentised residual and cook’s distance with one outlier +4SD removed. Graphs illustrating each ANOVA are presented in Fig. 4 a–f.

**Table 1:** Dependent variables analysed using ANOVA

ANOVA	Factor	DF	F score	Uncorrected *p* value	*p* value after Bonferroni^a^
MD width tooth #11	Bite type	1,29	60.50	< .001	< .01
	Position	2,58	20.51	< .001	< .01
	Bite × position	1.47,42.66^b^	13.84	< .001	< .01
Angle of rotation of tooth #11	Bite type	1,29	47.24	< .001	< .01
	Position	2,58	1.32	.274	ns
	Bite × position	2,58	6.80	.002	< .05
Inter-canine distance of the upper arch	Bite type	1,29	18.86	< .001	< .01
	Position	2,58	57.79	< .001	< .01
	Bite × position	2,58	17.84	< .001	< .01
MD width of tooth #41	Bite type	1,29	40.27	< .001	< .01
	Position	2,56^c^	7.16	.002	< .05
	Bite × position	2,56^c^	7.62	.001	< .01
Angle of rotation of tooth #41	Bite type	1,29	3.10	.089	ns
	Position	1.56,45.30^b^	4.86	0.011	ns
	Bite × position	2,58	9.72	< .001	< .01
Inter-canine distance of the lower arch	Bite type	1,29	92.27	< .001	< .01
	Position	2,58	39.81	< .001	< .01
	Bite × position	2,58	56.52	< .001	< .01

^a^6 ANOVAs were run so criterion p values were Bonferroni adjusted to .0083 for the < .05 threshold and .0017 for the < .01 threshold

^b^Greenhouse-Geisser corrected due to lack of sphericity

^c^A single outlier (+ 4SD) was removed to avoid biasing the model

The MD width of tooth #11 is observed in three different positions in the graph of Fig. 4 a for each method. For touch mark, there is a homogeneous decrease in the MD width compared with the true value as the estimated means at all positions fall below the reference line as opposed to the bite mark where there is an overall increase in the MD width, as the estimated means lie above the reference line.

Both marks display the completely opposite response for the angle of rotation of tooth #11 at all positions as shown in the graph of Fig. 4 b. The means for touch marks at all positions are below the reference line, which indicate a decrease in the angle of rotation as compared with the true value, whilst the means of bite mark at all positions lie completely on the opposite side of touch mark. This indicates that with change in position, bite mark shows an increase in their angle of rotation for tooth #11.

In the graph of Fig. 4 c, it can be observed that in case of touch mark, the inter-canine distance demonstrates a very varied response at all positions. At positions 1 and 3, the mean value falls above the reference line which indicates that lengthening of the upper arch occurs when teeth initially come in contact with the skin at these positions, whereas the touch marks created at position 2 showed a drastic decline in its dimensions as the means fall below the reference line.

This indicates that the change of position influences the inter-canine distance of the upper arch. Similarly, in the case of the bite mark, there is a substantial increase in the inter-canine distance at positions 1 and 3, which results in lengthening of the upper arch. But when the position of the arm was changed to position 2 there was a fall in the measurement of the inter-canine distance as the mean falls below the reference line. This indicates that there was a great degree of shrinkage seen in the dimension of the upper arch. The above observations indicate that the inter-canine distance of upper arch is influenced by the change in position of the arm. It can also be observed that when the bite mark was first created at position 1, there was a substantial degree of distortion present prior to the change in the position of the arm.

In the graph of Fig. 4 d, it can be observed that there is an increase in the measurements of the MD width of tooth #41 in case of touch marks and bite marks except in case of touch marks at position 1 where a slight decrease is seen. This means that when teeth initially come in contact with the skin at different positions, some degree of distortion in the MD width of tooth #41 is seen and in case of bite marks, as the position changes, some degree of distortion is seen in the MD width of tooth #41.

The graph shown in Fig. 4 e contrasts the behaviour for angle of rotation as observed for touch mark and bite mark at all positions. In the case of touch marks, a decrease in measurements is seen in at positions 1 and 2 as opposed to an increase in measurement at position 3. This indicates that the angle of rotation at positions 1 and 2 shows a similar behaviour when teeth initially come in contact with the skin as they tend to display a steeper angle of rotation as compared with the true value. Position 3 on the other hand displays an increase in the angle of rotation as compared with the other two positions, which indicates that at this position, the angle of rotation becomes flattered as compared with the true value. The exact opposite effect is seen in the case of a bite mark where there is a decrease in degrees at all positions, especially position 3.

In the graph shown in Fig. 4 f, the opposite responses of the inter-canine distance for touch marks and bite marks with change in position can be observed. In the case of touch marks, it can be observed that there is a decrease in the measurements of the inter-canine distance as compared with the true value, and in the case of bite marks, it can be observed that there is an increase in the measurements of the inter-canine distance as compared with the true value. This indicates that when teeth initially come in contact with the skin, shrinkage in the lower arch is observed and when a bite mark is produced in the skin, it results in lengthening of the inter-canine distance as there is a change in position.

## Discussion

The authors considered 1 mm as the threshold of significant distortion. The aim of this study was not to dictate the threshold of clinical significance for possible errors in bite mark analysis but to demonstrate the potential confounders in bite mark analysis on skin and changes in posture by confronting numerical results.

Analysing the results of ‘touch marks’ for upper arch, there was no statistically significant difference in the MD width of tooth #11 and inter-canine distance at all positions. A significant degree of distortion was observed in the angle of rotation of #11 at all positions. This indicates that on initial contact of teeth with the skin, there is distortion that occurs prior to the application of greater force to the skin and this affects the angle of tooth rotation greatly. This may be due to the elastic property of the skin. The human skin exists in a state of pretension which is greater in one direction than the other, and this relates to the anisotropic property of the skin [8, 13]. The tension is greater parallel to the tension lines but more relaxed perpendicular to the tension line. Therefore, the skin displays less extensibility parallel to the tension lines and more extensibility perpendicular to the tension lines [14].

Analysing the results of ‘touch marks’ for lower arch, there was also no statistically significant difference in MD width and the angle of rotation of tooth #41 and inter-canine distance at all positions. The small crown width might be a reason for the almost unchanged angle of tooth rotation. Overall, the range of means at position 1 lies between − 4.45 and 0.35, at position 2 lies between − 4.20 and 0.136, and at position 3 between − 2.65 and 0.16 for all the tooth and arch measurements (inter-canine distance). These values indicated that the skin tension varies within a single site, resulting in non-uniform values for the tooth and arch measurements. It can also be said that the degree of distortion also varies both within and between arches. Looking at the variation in the values for positions 2 and 3, it can be observed that the stiffness of the underlying tissue also tends to influence the shape and size of the mark, as the upper arm at position 2 was flexed and at position 3 was placed across the chest with the palm resting on the opposite shoulder.

Considering the results of ‘bite marks’ for the upper arch, a significant degree of distortion was observed when subjected to change in position. It can also be noticed that when the bite mark was initially created at position 1, there was already some degree of distortion seen prior to the change in position as there was change in the dimensions of the tooth and arch measurements whose means ranged between 0.37 and 4.06 mm. This indicates the non-linearity of the skin; that is, when pressure is applied on the skin, it enters the elastic phase where the rapid extension of the skin occurs and the elastin fibres tend to orient themselves in the direction of the force, but the extensibility of the elastic fibres is limited, so when the pressure applied is removed at a point where the fibres have reached their maximum elastic limit, the skin does not regain its original shape and some degree of distortion is seen. This is the property of hysteresis of the skin [8, 15]. Since the bite mark produced was parallel to the tension line, the bite mark displayed a ‘dragged’ appearance, and change in position of the arm to position 2 and position 3 further added to the degree of distortion. Significant distortion was also found in the lower teeth. Overall, the mean values for the tooth and arch measurements at position 2 ranged between − 0.47 and 4.38 mm, and at position 3, they ranged between − 2.68 and 3.62 mm. These variations in the mean values of the tooth and arch measurements in case of bite mark at all positions further support the fact that skin tension varies within a single site and between arches.

These results were consistent with previous studies of bite marks in human skin and how the biomechanical properties of the skin and the posture of the body further add to the distortion seen in the bite mark [13, 16, 17]. It is important to note that inter-canine distance has been used to differentiate the origin of the marks (human or animal) [18], to distinguish human adult bites (adult human; or small adult; child’s deciduous teeth) [19], and to estimate specific race and sex groupings by its relevance. It is important to note that warping, shrinkage, and distortion of the skin would make exact measurement of the inter-canine distance in a bite mark difficult [20]. A previous study investigated the size variation in bite marks produced by a single dentition, and the findings indicated that change in arch width is the predominant effect of distortion in skin [14].

Also, crown width marks registered on skin have been used in comparisons; therefore, measurements have been played an important role in the bite mark analysis and comparison, but the degrees of distortion should not be neglected. There is a great variation of anterior teeth crown width values in the literature, and the differences are reduced to millimetres. For instance, a study on white subjects found the crown width of central incisors ranging from 9.10 to 9.24 mm whilst the crown width of lateral incisors ranged from 7.07 to 7.38 mm [21]; therefore, the limitations of metric analysis start from the crown anatomy (incisal edge) and respective size range. Metric analyses on the skin are perhaps flawed as the distortion that has occurred can never be quantified in such resolution [22].

According to the ABFO (American Board of Forensic Odontology) *Standards and Guidelines for Evaluating Bitemarks* (revised in 2018), the dental characteristic is a feature or trait within a bite mark that represents an individual tooth variation and includes unusual wear pattern, notching, angulations, and fracture [23]. This study proved that the angulation of the tooth mark has been altered because of the skin and changes in posture; therefore, one might argue that angulation might not be a strong dental characteristic. The degrees of distortions present in a bite mark are variable and affect arch size and shape [6]. Another study proved that the uniqueness of the dentition cannot be perfectly transferred to the skin because the skin affects the ability to recognise unique dental features in a bite mark [24]. In addition, every occasion in which a dentition comes in contact with the skin can be considered a unique event [25].

Considering the admissibility of bite mark evidence, the ABFO no longer support the identification of individuals but if sufficient information is available to support conclusions, bite mark conclusions should only (a) *exclude* or (b) *not exclude* (*include*) *a dentition* [23]; however, the results of this study demonstrate that even exclusions might be invalid.

It is a common belief that all patterned injuries suspected of being human bite marks are compared with the dentitions of suspected biters, but most cases never get beyond the initial phase of analysis [26]. Comparison is more complex and there is a great number of important aspects to factor in by the expert. Reliability concerns only consistency of measurement but does not address whether a measurement is correct. Validity is concerned with the question of whether a measuring instrument (including opinions of humans) is generating correct answers; therefore, a number of forensic dentists might all agree on whether or not a suspect’s dentition made a bite mark (high reliability), but they might all be incorrect (low validity) [5].

An essential component of the determination of the validity of bite mark analysis is that a wide variety of techniques used in the physical comparison have been assessed and found valid [22]. Some might argue that the robustness of a technique is achieved by exposing its success rates, vulnerabilities, and potential error rates but there is no certainty that the usefulness of bite mark will be rebutted [27] due to disputable scientific foundations.

Limitations of the study included the unknown amount of force applied for creating the simulated bite mark and consequent lack of skin reaction. The degree of jaw opening whilst creating the marks was not measured because the bite registration should only show up to 1st or 2nd premolar.

## Conclusion

When touch marks were produced, there was distortion in one of the measurements on initial contact of the teeth with the skin even prior to any significant force being applied. In the case of a forced bite marks, a greater degree of distortion was detected, and this increased further when changing the position of the arm. The findings demonstrated that skin properties and posture influence distortion. This could lead to inaccurate measurements and misleading pattern interpretation of bite mark injuries.
